# Studies in Mouth Pathology from the Standpoint of Toxin Absorption and Its Sequlae*Reprinted in abstract from the Pacific Dental Gazette, of September, 1915.

**Published:** 1915-10

**Authors:** Julio Endelman, James D. McCoy


### STUDIES IN MOUTH PATHOLOGY FROM THE STANDPOINT OF TOXIN ABSORPTION AND ITS SEQULAE.*

BY DOCTORS JULIO ENDELMAN AND JAMES D. MCCOY.

As teachers in the College of Dentistry of Southern California University, we have for several years endeavored to impress upon students the significance of pathology in clinical dentistry, and to impress upon them that the value of pathology as a practice asset in the performance of the score or more of operations looked upon by most students in the light of mechanical procedures rather than of therapeutic measures to be undertaken and carried out upon a rational basis—rationalism in this connection having reference to an understanding of disease etiology, disease evolution and disease prevention.

May we be permitted to reiterate the statement that the practitioner who in his work limits himself to technical procedures only and disregards through disinclination or insufficient training normal and pathologic physiology is doing a gross injustice to the profession to which he has sworn tacit allegiance. The ability to successfully cope with problems involving the display of finger skill is in our profession a *sine qua non*, but operations of this nature carried out without due regard to the physiologic and pathologic phenomena involved, not only from the standpoint of eradication, but likewise from that of disease prevention are eventually to be judged on the basis of failure, either partial or total. These failures may not, however, be in the nature of a dislodged prosthetic appliance or a faulty filling but in the abnormal reactions

---

* Reprinted in abstract from the Pacific Dental Gazette, of September, 1915.

which reduce the usefulness of the individual and in some instances lead to invalidism and perhaps death.

We do not look upon the study of pathology, general or special, in the light of a cultural professional asset, but from the viewpoint of a basal rock of the professional superstructure. It matters not whether we direct our attention and endeavor along lines of specialism, or whether we devote ourselves to operations in the field of general practice, the value of dental pathology in every division and subdivision of the science and art of dentistry is an indispensable requisite and the only means of divorcing our profession from the intense empiricism to which unfortunately it has been wedded for so many years.

Pathology being the very reason for the existence of men supposedly versed in the science of treatment, is it not paradoxical that this division of dental knowledge should not be in that state of advancement so noticeable in other departments of the dental curriculum?

The rescuing of dentistry from the realm of a mechanic art and the maintaining of a place for it among the learned professions rests to a large extent upon the degree of progress to be attained along lines of pathology and of therapeutics and realizing to the fullest extent the deficiency of information in these fundamental departments of dentistry, we are constrained to urge further investigations in the field of disease incidence, disease evolution and disease prevention.

The greater porton of this paper will deal with phases of general and dental pathology in which the pulp is the field of disease evolution and therefore we trust the digression to which we have already referred will be considered permissible and not irrelevant.

Pulp infection whether of a source extrinsic or intrinsic to the tooth is in, by far, the largest number of cases the direct source of pericemental infection and consequently in tracing back the course of disturbances in the pericemental tissues our studies have led us into a somewhat minute consideration of the pulp, its diseases, usual methods of treatment in addition to a consideration of the areas around the roots of teeth which in the light of our studies become the starting point of innumerable forms of intoxications in the more deeply situated structures and of reflex manifestations in which the factor pain is present in varying degrees of intensity.

While it has been impossible to ascertain the exact nature of the mechanical and therapeutic procedures that were followed in the cases with remote pathologic manifestations that we have studied, we have, however, been able to reach a main conclusion, namely that the technique of pulp extirpation, subsequent treatment, or the methods in vogue of treating infected root canals or septic pericemental inflammation, are not uniformly successful within such limits as any therapeutic or surgical procedure should be expected to be. And if we imply inefficient technique let us make it as emphatic as we possibly can that we have no charge to make of either incompetency or carelessness. The cases that come under the inspection of pathologist and radiologist arise from every sphere of the profession, but mainly from practitioners of well founded honorable standing in the profession and outside of its border lines.

Whether it be because the pathology of the dental pulp remains in a confusing state and that of the pericemental membrane is likewise as yet at some distance from the goal of definiteness, or whether because of insufficiency in methods of diagnosis, we are unable to say, but we must again record the fact that many among the cases that have come under our observation were permitted to go along untreated for months and years, not only without suspicion of any existing pathologic state in the jaws; but with assurances that the teeth and supporting tissues were in the best of conditions.

The processes of putrefaction and suppuration of the dental pulp for instance are two phases of the subject which we are inclined to believe are in need of revision with the end in view of ascertaining, if possible, what means, therapeutic or mechanic, may be instituted toward the eradication of the disturbances in surrounding deeper structures to which they give rise and in conformity with such additional pathologic data as might be added to the sum total of information available upon these topics at the present time. We are satisfied that our clinical observations show that at least in the proportion of cases examined by us methods of pulp and pericemental therapeutics generally practiced are inefficient and we venture to believe that conditions do not differ in the profession at large.

One of the difficulties encountered in the endeavor to study the degree of relative efficiency of the various methods of treatment of infectious conditions of root canals and peri-apical tissues is to be found in the remoteness, as to time, of the manifestations of insufficient therapeusis.

Many a tooth is to all intents and purposes successfully treated, conditions in the root canals appear favorable, the tooth does not react to percussion, all visible and subjective symptoms are favorable and the operator proceeds to fill the canal or canals and to insert a filling of the permanent type, thereby placing the seal of finality upon his work. Months and years may follow without there appearing any sign of danger and still the peri-apical tissue may have been right along the seat of an inflammatory septic process which almost invariably gives rise to manifestations of general toxemia, perhaps of a very mild nature.

The futility of attempting to obliterate areas of septic inflammation of the pericemental membrane of decided chronicity, has become patent to us in numerous cases and our convictions of wrong-doing by withholding advice to that effect from patients in whom the systemic manifestations of these foci of infection in the jaws are definitely established, is well-nigh positive.

We wish to counteract any impression our statements might produce in the minds of some, perhaps to the effect that we have degenerated into worshippers at the shrine of the steel forceps. We condemn wholesale extractions as determinedly as we ever have heretofore, and do not avail ourselves of this otherwise efficient curative method of all dental ills, except as a measure of last resort, but of last resort we consider it to be in the kind of cases to which we have alluded, reserving the privilege of amending our views in the event of the discovery of therapeutic methods of equal efficiency and thoroughness.

The presence of septic phenomena in and around pericemental tissues gives rise to degenerative changes in the surrounding osseous tissues which again, regardless of the bactericidal effects of therapeutic measures, we are unable to correct by methods from which surgical procedures are eliminated. These complications which partake of the character of a septic rarefying osteitis are beyond the possibilities of therapeutic medication and require that they be combated by surgical procedures.

Rarefying septic osteitis is a pathologic entity very frequently encountered within the alveolar walls and in the tissues of the jaw and is verily the only available index, at the present time, of the degrees of intensity and chronicity of septic processes arising from foci of infection in root canals and pericemental structures.

A rarefying osteitis from a pathologic standpoint is a gradual degeneration terminating in death of the cellular elements of bone, the outcome of a bacterial invasion and perhaps the filling up the cracks, crevices and deficiencies in the bony structure by young cellular forms of connective tissue.

The pathologic facts to which we have so far referred may be summarized as follows:

1. The so-called chronic dento-alveolar abscess is a combination of reactions to bacterial infection comprising changes in the pericemental membrane (destructive inflammation), in the cementum (limited necrosis), and in the alveolar tissues (rarefying or proliferating osteitis).

2. Septic infection of the pericemental membrane when of the chronic type may give rise to cyst formation or to proliferations of epithelial debris in the parenchyma of the pericemental membrane.

3. Many cases of persistent inflammation are due to the presence of bacterial exciters within cystic formations or within masses of epithelial and fixed tissue cells.

4. Cases of pericemental infection which resist therapeutic treatment for a period of time varying from four to six weeks are in the majority of cases of the cystic type or of the type in which the epithelial debris and the fixed tissue cells have proliferated (fibrous pericemental degeneration).

5. The diagnosis of a large number of cases of septic inflammation of the pericemental membrane leading to toxin absorption and accompanied by osteitis of the rarefying or proliferating type is impossible without the assistance of the X-ray.

6. Teeth, the roots of which have been treated and filled and upon which areas of septic infection are to be found by means of radiographic diagnosis, should be treated surgically, either by amputating the root apices or by extracting the offending organs.

7. Such conditions as rarefying osteitis of septic origin in the proximity to the tooth root which has been the seat of a septic process of marked chronicity should not be permitted to go untreated. The curetment of these areas is recommended. The extraction of the tooth *per se* is not sufficient to bring about the restoration of the tissues to a condition of health.

CASE NO. 1.

Woman aged thirty-six who had previously been in apparent good health complained of acute pain in her face. In addition, she complained of a series of symptoms typical of general toxemia. She seemed anemic and decidedly below par. She sought the services of a dentist for the purpose of ascertaining whether any abnormality could be detected in her teeth that would account for the reflex pain from which she had been suffering for some time past. She was referred to us for a radiograph. An area of osteoporosis was found around the root of the second lower bicuspid indicative of chronic septis in the pericemental tissues and within the bony structures. The tooth had been previously treated and the root canal carefully filled. The bicuspid was extracted and almost immediately the pain and toxic symptoms began to improve and have continued to do so uninterruptedly.

CASE NO. 2.

Man aged forty-five who gave a history of continued good health up to the time of the incidence of the symptoms which led to his being placed under our observation. He had evidently been a man of vigorous constitution and non-sedentary in his habits. He complained of lack of appetite, lassitude, inability to exert himself to any extent without experiencing a sense of fatigue. The radiograph showed a marked chronic septic condition under the roots of the lower second molar, which tooth evidently had been treated years previously and in which the root canal had not been completely filled. There was a tendency to percussion in several teeth and consequently a definite diagnosis could not be made at the time. The radiograph came to our assistance, the offending tooth was extracted, and a complete recovery followed within a period of three or four weeks.

### CASE NO. 3.

Man aged about sixty years. This case seems to us to present features of special interest because the septic conditions about the roots of some of the teeth were evidently responsible for an interruption in the mental balance of the patient. The man, of limited means, had endeavored for some time past to secure a berth in an old man's home but on account of the mental and physical symptoms the authorities of the institution rejected his application. He was referred to us by his physician to ascertain whether any abnormality could exist in his jaws and around his teeth which could be made to account for the toxic symptoms from which he was suffering, and also for the disturbance in psychic function. We found what would warrant us in assuming that the foci of infection in his jaws were at least a factor in the production of the pathologic symptoms herein described. On the left side we found conditions as follows:

A chronic abscess and cystic formation around the roots of the lower first bicuspid; an incarcerated root in the upper jaw mesial to the second molar; also foci of infection in the distal root of the lower third molar, above and around the roots of the upper second bicuspid and upper third molar. On the right side a number of foci of infection are to be detected in the lower and in the upper jaws. All the teeth that were considered responsible for the conditions herein described were extracted and the improvement that followed was so marked that he was permitted to become a member of the household in the institution for the aged, to which admission had been denied him some months previously.

CASE NO. 4.

Woman aged about thirty giving a history of rheumatoid arthritis, with marked pain in the region of the shoulder blades; pseudo-ankylosis; marked anemia, fatigue, lassitude, etc. This case also exhibited marked reflex nervous manifestations. From this case streptococcus veridens was isolated. The tooth that was considered beyond therapeutic measures, *i. e.*, the lower left second molar, was extracted; some of the other teeth, the seat of a chronic septic inflammation, were treated in the approved ways while the apices of the single-rooted teeth were amputated. The improvement which has followed is most encouraging.

CASE NO. 5.

Man aged fifty years complained of pain in the shoulder on the same side as that upon which was found to exist a chronic septic focus around the apical third of the lower bicuspid tooth. Removal of the tooth was followed by complete recovery. You will kindly notice in this case that slight drainage existed along the distal side of the root, but, however, not sufficient to prevent toxin absorption.

CASE NO. 6.

Woman aged about forty giving a history of no pain but on the other hand one of disturbance of nutrition accompanied by anemia. Being the wife of a physician, her case was studied with care by a number of medical practitioners, all of whom reported absence of visceral lesion. Several foci of infection were found in the mouth, namely, one around the roots of the lower first bicuspid, one around the roots of the lower right second molar, and one on the distal side of the upper right second bicuspid. Eradication of these areas was followed by decided improvement.

CASE NO. 7.

Young woman twenty years of age who for a period of several months had been suffering intense pain. She had sustained a loss in weight of sixteen pounds in six weeks. This case showed impaction of the upper and lower third molar. Their removal was followed by severe post-operative pain, but three weeks after she had regained her usual normal good health.

CASE NO. 8.

Woman aged thirty years. This patient exhibited symptoms of intense toxemia for a number of months. Prior to the onset of these symptoms she reported having been in good health. She was referred to us to ascertain whether the condition of the lower first molar, which had been treated and the root canal filled, was satisfactory. Suspicion had been aroused because the tooth was slightly tender to percussion. While a slight abnormality is detected, the main difficulty was, however, in the second bicuspid. This patient was in such an exhausted condition as to require anywhere from sixteen to twenty-four hours sleep per day, and even after such a long period of quietness she would awake with a feeling of fatigue as intense as it seemed to be at the time of retiring. The lower second bicuspid was extracted and in the course of a few weeks the symptoms entirely disappeared and has since reported that eight hours sleep satisfy her completely.

CASE NO. 9.

Man aged about forty-five giving a history of lameness in the right shoulder, torticolis and continued toxin absorption. He did not have to state that he was a sick man—that was self-evident. The roots of the first molar show an intensified septic process. The tooth was extracted and was followed in due time by great improvement. The history of this case has not been followed up since the first marked improvement occurred.

CASE NO. 10.

Man aged about forty-five years with symptoms of toxin absorption and slight pain on the left side of the mandible. A large septic focus was found between and below the roots of the lower first molar. Extraction was followed by recovery.

CASE NO. 11.

Man aged thirty-five had a bridge inserted six years previously. A pathological proliferating process is going on in the body of the mandible doubtless caused from irritation by a septic pulp contents of the lower right first molar. Clinical symptoms, together with this radiograph, would warrant the establishment of a diagnosis of osteosarcoma.

The series of cases which we will now endeavor to describe belong to a class in which the presence of abnormal degrees of irritation in the jaws bring about severe pain manifestations, reflex in character. The conditions encountered in these cases indirectly undermine the individual's health because continued pain acts as a powerful source of inhibition of those nervous stimuli, which are indispensable for the continuance of all vital functions. Pain, regardless of its source, gives rise to functional disturbances proportionate to its degree of severity. Pain must be combated without delay because of its injurious effect upon the nervous function. So well did Souilier, that scholarly therapeutist of Lyons, appreciate the significance of disturbed functional activity caused by continued degrees of pain, that he referred to this sympton as being to the nervous system what hemorrhage is to the vascular system.

CASE NO. 12.

Woman aged sixty years had supposedly all teeth extracted seventeen years ago. Since that time has been wearing artificial dentures. The pain in her face was paroxysmal at short intervals, and was accompanied by muscular twitchings typical of tic douloreux. The radiograph shows an erupted lower third molar. The tooth was extracted and within a few days the so-called tic douloreux disappeared.

CASE NO. 13.

Man aged about thirty-five giving a history of tic douloreaux of fifteen years standing. The X-ray shows an impacted lower third molar on the right side which was removed. Improvement has followed but the case as yet is far from being cured.

CASE NO. 14.

Man aged thirty-eight giving a history of pain at times dull, at times acute, lasting for about a week or ten days. At times there would be no pain for several months.

Pain radiating to the eyes and ears and arising from the teeth. Tentative diagnosis of incarcerated supernumerary tooth.

CASE NO. 15.

This case gave a history of intense pain in the right ear of ten weeks' standing. The specialist to whom she was referred treated her for inflammation of the middle ear. As a measure of last resort and in order to relieve the intensity of her suffering a puncture of the drum was made, but without bringing about the much-sought-for relief. The removal of an impacted right third molar brought about complete cessation of all symptoms in one week's time.

CASE NO. 16.

Girl aged seventeen years with painful reflexes in the ear. Removal of an unerupted third molar resulted in a cure within six weeks' time.

CASE NO. 17.

Man aged about forty years giving a history of excruciating pain on the right side of the face. His dentist suspected the third molar, which diagnosis was proven to be incorrect. At the time of the taking of the radiograph the man had not slept for eight consecutive nights. A marked infection around the roots of the lower third molar and osteo-porosis near the mandibular angle were found. The extraction of the tooth, eradication of the area of partial bone necrosis and subsequent post-operative treatment was followed by complete cure.

CASE NO. 18.

This patient complained of intense pain in the eyes and in the ear and gave a negative history of painful symptoms in the teeth. This young girl also exhibited intense mental and hysterical symptoms. This pain had been going on for a period of three years. The radiograph showed an impacted upper and lower third molars on both sides. The teeth were extracted, and while there was marked post-operative disturbances, eventually she regained her normal health.

CASE NO. 19.

Woman aged thirty-two, school teacher, giving a history of intensified painful facial reflexes. Foci of infection around the roots of the lower right first molar and mandibular periostitis in an area immediately below the affected tooth was the diagnosis established by means of the X-ray. Removal of this tooth and surgical treatment of the osseous region was followed by a cure.

CASE NO. 20.

Girl aged nineteen years with painful reflexes localized in the ear. Upper and lower impacted third molars were extracted and recovery soon followed.

CASE NO. 21.

Girl aged seventeen giving a history of severe facial pain. Radiograph shows a triple impaction in the lower jaw. These teeth eventually erupted of their own accord.

CASE NO. 22.

Man about thirty years of age giving a history of severe facial pain. A fistula was found on the mandibular ridge, which led to the suspicion that a root remained in that section of the jaw. The radiograph, however, shows an unerupted first molar tooth which was extracted, the operation being followed in due time by complete disappearance of all symptoms.

CASE NO. 23.

Man aged about thirty years giving a history of reflex pain in the face, intermittent in character. The lower molars were found to be the seat of septic processes and an impacted lower third molar was discovered. The molars were treated, the impacted tooth was removed and improvement followed within a few weeks.

CASE NO. 24.

Boy aged nine years presenting a bulging upon the anterior surface of the mandible. Several impacted teeth were found which were doubtless accountable for the presence of the osteitis of the proliferating type.

CASE NO. 25.

Woman aged forty-five years giving a history of recurrent dull pain on the right side of the face in the upper and lower teeth. The radiograph shows an impacted lower third molar which was extracted and which was followed by complete disappearance of the pain manifestations. As a side light on the influence of pathological condition of the teeth upon phonation, this patient was of peculiar interest to us. When she called for advice and treatment her voice was of a deep, sonorous, husky type. With the extraction of the offending tooth, the charm of feminine voice returned.

CASE NO. 26.

Man aged twenty-nine giving a history of general sinusitis of five years' standing. Two years ago he was operated on for ethmoidal-sinusitis but without satisfactory results. He reported having been free from all trouble in his mouth up to the time of the insertion of the bridge shown on the radiograph. A septic condition was found over the root of the first bicuspid, which, by the way, occupies an area in close proximity to the root of an encysted cuspid, which we inverted, the incisal edge pointing upwards. The radiograph shows conditions after the removal of the abscessed tooth, the patient making marked improvement. Doubtless in this case the infection in the antrum was due to the spreading of the septic process around the root of the second bicuspid to the antral muco-periosteum. The infection in the other sinuses of the face was a continuation of the infection in the antrum.

### CASE NO. 27.

This patient gave a history of reflex painful manifestations in the face and stiffness of the neck. Radiographic diagnosis shows a portion of the root of a tooth which had been supposedly extracted one year previously. The removal of the root corrected the difficulty.

### CASE NO. 28.

Man aged about forty-five years giving a history of stomach trouble. He had been through a detailed physical examination which revealed nothing, the physician pronouncing the man, as far as he could ascertain, absolutely normal. He referred him to a dentist because of the possibility of septic foci in the region to which might be traced the systemic symptoms of rheumatoid arthritis in the shoulder and in the lower limbs. The crowned teeth in the lower jaw show areas of marked septic inflammation. They were treated for a while, but finding that they did not respond to therapeutic measures, the teeth have since been extracted. This case is one which has come under our observation very recently and so far we are unable to report any marked improvement, although some slight abeyance of the symptoms is noticeable.

